# Characterization of the mutational landscapes in Japanese patients with early-onset colorectal cancer from comprehensive genomic profiling data

**DOI:** 10.1007/s10147-025-02889-w

**Published:** 2025-10-11

**Authors:** Yutaka Okagawa, Tomohiro Kubo, Shin Ariga, Norito Suzuki, Hiroki Tanabe, Susumu Sogabe, Atsushi Ishiguro, Tatsuru Ikeda, Shinya Minami, Masahiro Hirakawa, Ichiro Kinoshita, Kohichi Takada

**Affiliations:** 1 Division of Medical Oncology, Department of Internal Medicine, Sapporo Medical University School of Medicine, South 1, West 16, Chuo-ku, Sapporo, Hokkaido 060-8543 Japan; 2 Department of Medical Oncology, Hokkaido University Hospital, Sapporo, Hokkaido Japan; 3 Division of Gastroenterology, Department of Medicine, Asahikawa Medical University, Asahikawa, Hokkaido Japan; 4 Department of Medical Oncology, KKR Sapporo Medical Center, Sapporo, Hokkaido Japan; 5 Department of Medical Oncology, Teine Keijinkai Hospital, Sapporo, Hokkaido Japan; 6 Department of Cancer Genome Medical Center, Hakodate Goryoukaku Hospital, Hakodate, Hokkaido Japan; 7 Department of Gastroenterology, Oji General Hospital, Tomakomai, Hokkaido Japan; 8 Division of Clinical Cancer Genomics, Hokkaido University Hospital, Sapporo, Hokkaido Japan

**Keywords:** Colorectal cancer, Early-onset, Japanese, Genomic alteration, Comprehensive genomic profiling

## Abstract

**Background:**

The incidence of early-onset colorectal cancer (EoCRC), defined as a CRC diagnosed in individuals younger than 50 years, has been increasing globally. The clinicopathological differences between EoCRC and late-onset CRC (LoCRC: diagnosed in individuals older than 50 years) are suggestive of distinct genomic landscapes. The aim of this study was to assess the differences in genomic alterations in Japanese patients with EoCRC and LoCRC from multiple institutions across Hokkaido using comprehensive genomic profiling data.

**Methods:**

The patient’s background, CRC location, pathological findings, clinical stage at presentation, prognosis, and genomic alterations of the EoCRC and LoCRC groups were compared.

**Results:**

A total of 317 CRC patients were analyzed, including 61 with EoCRC and 256 with LoCRC. Right-sided CRC and differentiated histology were significantly less common in the EoCRC group. There was no significant difference in the median survival duration between the two groups. Genomic profiling revealed significantly higher frequency of *SMAD4*, *FLT3*, and *CDK8* alterations in EoCRC patients compared to LoCRC patients (*p* = 0.016, *p* = 0.023, and *p* = 0.035, respectively). Cell cycle pathway alterations were also significantly enriched in the EoCRC group (*p* = 0.003). Additionally, *SMAD4* mutations were associated with poor prognosis in both groups.

**Conclusions:**

*SMAD4*, *FLT3*, and *CDK8* alterations were significantly more prevalent in EoCRC patients, suggesting that these genes likely contribute to the distinct molecular pathogenesis of EoCRC, and may also serve as potential therapeutic targets. Further studies are warranted to elucidate their biological significance and explore their potential in the development of targeted therapies for Japanese patients with EoCRC.

**Supplementary Information:**

The online version contains supplementary material available at 10.1007/s10147-025-02889-w.

## Introduction

The incidence of early-onset colorectal cancer (EoCRC), defined as a CRC diagnosed before the age of 50, has risen globally in recent years [1–3]. Various risk factors have been implicated in the development of EoCRC, such as increased consumption of Western diet, changes in the gut microbiome, alcohol consumption, obesity, genetics, and racial disparities [3–8]. However, the precise mechanisms underlying the pathogenesis of EoCRC remain unclear. EoCRC is often occurs in individuals without a family history of CRC. It frequently arises in the rectum, and is characterized by a higher proportion of poorly differentiated histology, including signet-ring cell carcinoma and mucinous carcinoma [9–12]. Moreover, EoCRC is more likely to be diagnosed at an advanced stage compared to late-onset CRC (LoCRC), which afflicts those aged 50 years or older [10]. While the prognosis for EoCRC is similar to that of LoCRC [13], the increasing incidence of EoCRC is a global public health concern given the profound social and personal impact of cancer diagnosis at a relatively young age. Limited studies have been conducted on EoCRC patients in Japan [14, 15], even though the age-standardized prevalence rate of EoCRC has been rising in the Japanese population [16], and this trend is expected to continue.

A key molecular driver of cancer is the accumulation of genomic and epigenomic alterations. The distinct clinicopathological features of EoCRC and LoCRC suggest differences in genomic alterations as well. Comprehensive cancer genomic profiling (CGP) has enabled systemic identification of genomic alterations in cancer patients, and several studies have assessed the genomic alterations between EoCRC and LoCRC using this approach [17, 18]. Li et al. [17] analyzed the CGP results of CRC patients in China, and identified significantly more genomic alterations in EoCRC, including those in *PTCH1*, *KMT2A*, and *B2M*, compared to LoCRC. Busico et al. [18] analyzed the CGP data of an Italian CRC cohort, and found that *RNF43* gene mutations were more prevalent in the pediatrics patients, while the frequency of *APC* mutations increased with age. Recent studies have also highlighted racial differences in the genomic landscape of various cancers [19].

The aim of this study was to identify the differences in genomic alterations between Japanese EoCRC and LoCRC patients on the basis of CGP data, and provide insights that will contribute to the development of optimal therapeutic strategies for EoCRC patients.

## Patients and methods

### Study design and patients

The CGP data of advanced CRC patients from multiple centers across Hokkaido, Japan was retrospectively analyzed. Japanese CRC patients who underwent CGP with FoundationOne^®^ CDx genome profiling (F1CDx; Chugai Pharmaceutical, Tokyo, Japan), FoundationOne^®^ Liquid CDx genome profiling (F1LCDx; Chugai Pharmaceutical), OncoGuide™ NCC Oncopanel System (NCC Oncopanel; Sysmex Corporation, Kobe, Japan), Guardant360^®^ CDx (Guardant; Guardant Health Japan Corp, Tokyo, Japan), and GenMineTOP^®^ Cancer Genome Profiling System (GenMineTOP; Konicaminolta, Tokyo, Japan) between June 2019 and October 2024 were consecutively enrolled. The inclusion criterion was a pathological diagnosis of CRC, including patients with unresectable stage IV disease and patients with stage I–III or stage IV disease who developed postoperative recurrence. Patients with congenital anomalies, familial adenomatous polyposis, or inflammatory bowel diseases, or those who refused to provide written informed consent were excluded. The study was conducted as per the guidelines of World Medical Association Declaration of Helsinki, and written informed consent was obtained from all patients.

### Genomic analysis

According to a previous report [20], genomic alterations were classified into seven tiers (A to F, and R) of evidence-level classifications. Actionable genomic alterations were defined as alterations at or above evidence level D as previously described [21]. Variants of unknown significance were excluded from the analysis in this study. Signaling pathways were classified according to the Japanese version of the Cancer Genomic Atlas [22].

### Outcome measures

The patients were categorized into the EoCRC and LoCRC groups depending on the age at diagnosis. The patient’s background, CRC location, pathological findings, clinical stages at presentation, prognosis, and genomic alterations of the two groups were compared. A family history of CRC was defined as positive CRC diagnosis in a first-degree relative. Based on the predominant histopathological features, the CRC cases were classified as differentiated-type (well differentiated, moderately differentiated, and papillary adenocarcinoma) or undifferentiated-type (poorly differentiated, mucinous adenocarcinoma, and signet-ring cell carcinoma) based on the predominant histology. Clinical stages were classified according to the Union for International Cancer Control (UICC) TNM version 8. Prognosis was evaluated on the basis of overall survival (OS), which was calculated from the date of diagnosis of an unresectable cancer or recurrence until death.

### Statistical analysis

All statistical analyses were performed using EZR (Saitama Medical Center, Jichi Medical University, Saitama, Japan), a graphical user interface for R version 2.13.0 (R Foundation for Statistical Computing, Vienna, Austria) [23]. Quantitative variables were expressed as median, and categorical variables as absolute numbers and percentages. Pearson’s Chi-squared test and Mann–Whitney *U*-test were applied as appropriate. Survival rates were calculated using the Kaplan–Meier method and compared by the log-rank test. A *p*-value of < 0.05 was considered statistically significant.

## Results

### Patient characteristics and clinicopathological features

A total of 321 patients with CRC were initially enrolled, and four patients were excluded due to congenital anomalies (*n* = 1), familial adenomatous polyposis (*n* = 1), and inflammatory bowel diseases (*n* = 2). Among the remaining 317 patients, 61 (19.2%) were categorized into the EoCRC group and 256 (80.8%) into the LoCRC group (Table 1). The median age at CRC diagnosis was 45 years in the EoCRC group and 64 years in the LoCRC group. The gender distribution was similar in both groups. Similarly, there were no significant differences in the proportion of patients with a medical history of cancer excluding CRC or a family history of CRC (11.5% in the EoCRC group vs. 15.2% in the LoCRC group). Right-sided CRC was less common among the EoCRC patients compared to the LoCRC patients (8.2% vs. 26.2%, *p* = 0.003), and the proportion of patients with predominantly differentiated-type CRC was significantly lower in the EoCRC group than in the LoCRC group (83.6% vs. 92.6%, *p* = 0.029). On the other hand, the percentage of patients initially diagnosed with Stage IV disease was similar in both groups (55.7% in the EoCRC vs. 66.8% in the LoCRC group). There was also no significant difference between the two groups regarding the CGP testing methods or tumor sampling sites. The median survival duration was 41 months in the EoCRC group and 37 months in the LoCRC group, and the difference was not statistically significant (*p* = 0.978) (Supplementary Fig. 1).

**Table 1:** Patient characteristics and clinicopathological features

	EoCRC	LoCRC	*p* value
	*n* = 61	*n* = 256	
Sex (M:F)	30:31	137:119	0.542
Past history of malignancy	3	25	0.231
Family history of CRC	7	39	0.455
Location			
Right-side	5	67	0.003
Left-side	23	77	0.250
Rectum	33	112	0.145
Histology			
Differentiated	51	237	0.029
Undifferentiated	10	13	0.002
Others	0	6	0.228
Initial stage			
I	1	4	0.966
II	2	14	0.483
III	24	67	0.041
IV	34	171	0.105
Sampling sites			
Primary tumor	32	163	0.106
Metastatic lesions	24	71	0.076
Blood	5	22	0.921
Comprehensive genomic profiling			
FoundationOne CDx	51	194	0.191
FoundationOne Liquid	4	18	0.896
OncoGuide NCC	5	31	0.387
Guardant360CDx	1	4	0.966
GenMineTOP	0	9	0.138

*CRC* colorectal cancer, *EoCRC* early-onset colorectal cancer, *LoCRC* late-onset colorectal cancer

### Analysis of genomic landscape

**Fig. 1:**
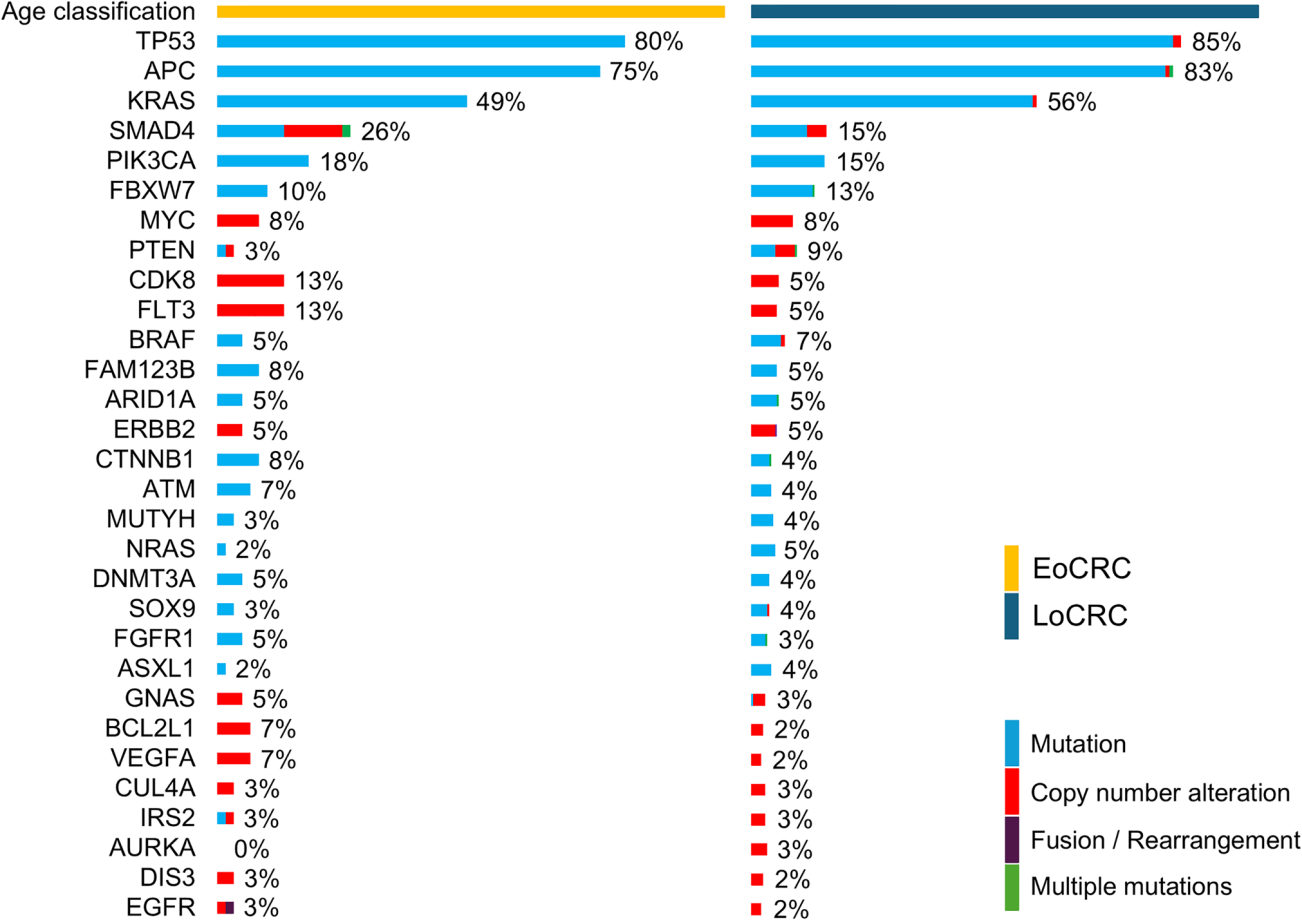
Genomic alterations in EoCRC and LoCRC patients. The top of 30 genomic alterations in the overall CRC cohort are shown, with the type of each alteration indicated

**Fig. 2:**
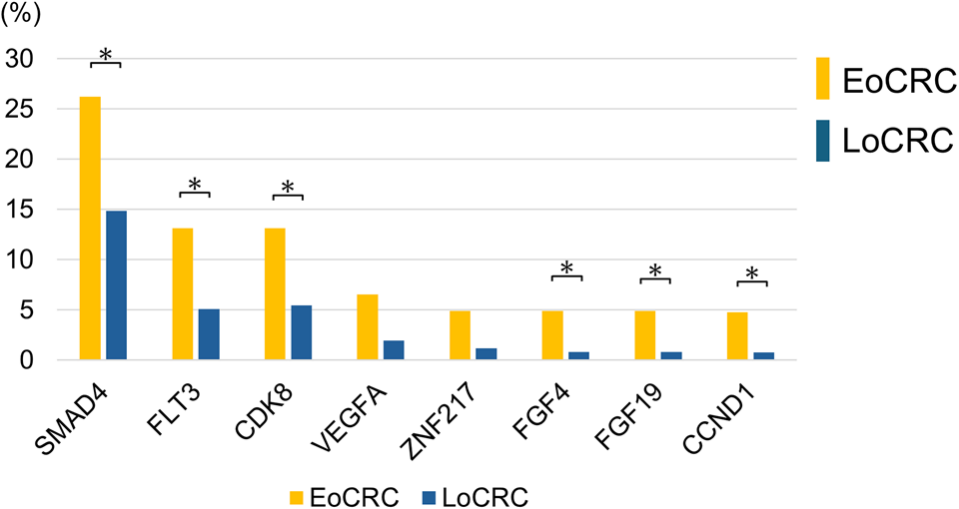
Differences in genomic alterations between EoCRC and LoCRC groups. Comparison of the frequency of specific genomic alterations between EoCRC and LoCRC groups

Four patients in this cohort had no detectable genomic alterations. The most frequently observed alterations were in *tumor protein p53* (*TP53*: 83.9%), *adenomatous polyposis coli* (*APC*: 81.7%), and *Kirsten rat sarcoma virus* (*KRAS*: 54.9%). The genomic alterations in the EoCRC and LoCRC groups have been depicted in Fig. 1, and Fig. 2 shows the genes with relatively greater differences in alteration frequency between the two groups. Alterations in the *mothers against decapentaplegic homolog 4* (*SMAD4*) (*p* = 0.016), *FMS-like tyrosine kinase 3* (*FLT3*) (*p* = 0.023), and *cyclin-dependent kinase 8* (*CDK8*) (*p* = 0.035) genes were more prevalent in the EoCRC group compared to the LoCRC group. In addition, fibroblast growth factor 4 (FGF4) (*p* = 0.02), FGF19 (*p* = 0.02), and cyclin D1 (CCND1) (*p* = 0.02) were also more frequently altered in the EoCRC group. Alterations in *vascular endothelial growth factor A* (*VEGFA*) (*p* = 0.052) and *zinc finger protein 217* (*ZNF217*) (*p* = 0.053) also tended to be more common in the EoCRC group, albeit without statistical significance. Nevertheless, the mean number of actionable genomic alterations in the EoCRC and LoCRC groups were similar (5 vs. 6, *p* = 0.099). Microsatellite instability-high (MSI-high) was detected in two EoCRC patients (3.3%) and two LoCRC patients (0.8%). Furthermore, two patients (3.3%) in the EoCRC group and 15 patients (5.9%) in the LoCRC group showed high tumor mutation burden (TMB-high). The differences were not significant.

### Pathway alteration analysis

**Fig. 3:**
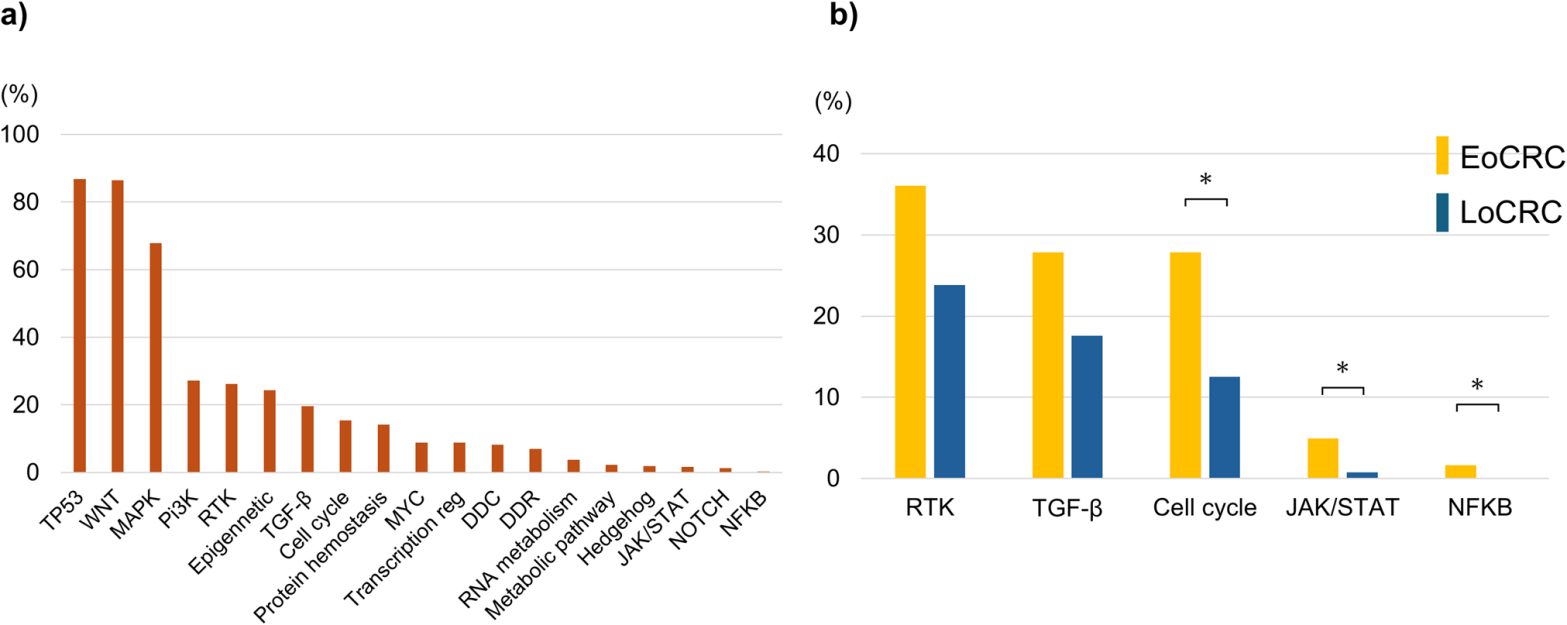
Signaling pathway alterations in CRC. **a** Distribution of altered signaling pathways in the overall cohort. **b** Comparison of pathway alterations between EoCRC and LoCRC groups

The TP53 (86.8%), WNT (86.4%), and MAPK (67.8%) pathways were the most commonly altered signaling pathways in the entire cohort (Fig. 3a). As shown in Fig. 3b, cell cycle pathway (27.9% vs. 12.5%, *p* = 0.003), JAK/STAT and NF-κB signaling pathway were more frequently altered in the EoCRC group compared to the LoCRC group. Although the receptor tyrosine kinase (RTK) and TGF-β signaling pathways were also more commonly altered in the EoCRC (RTK: 36.1%, TGF-β: 27.9%) group than in the LoCRC group (RTK: 23.8%, TGF-β: 17.6%), the difference was not significant.

### *SMAD4* and *FLT3* alterations

**Fig. 4:**
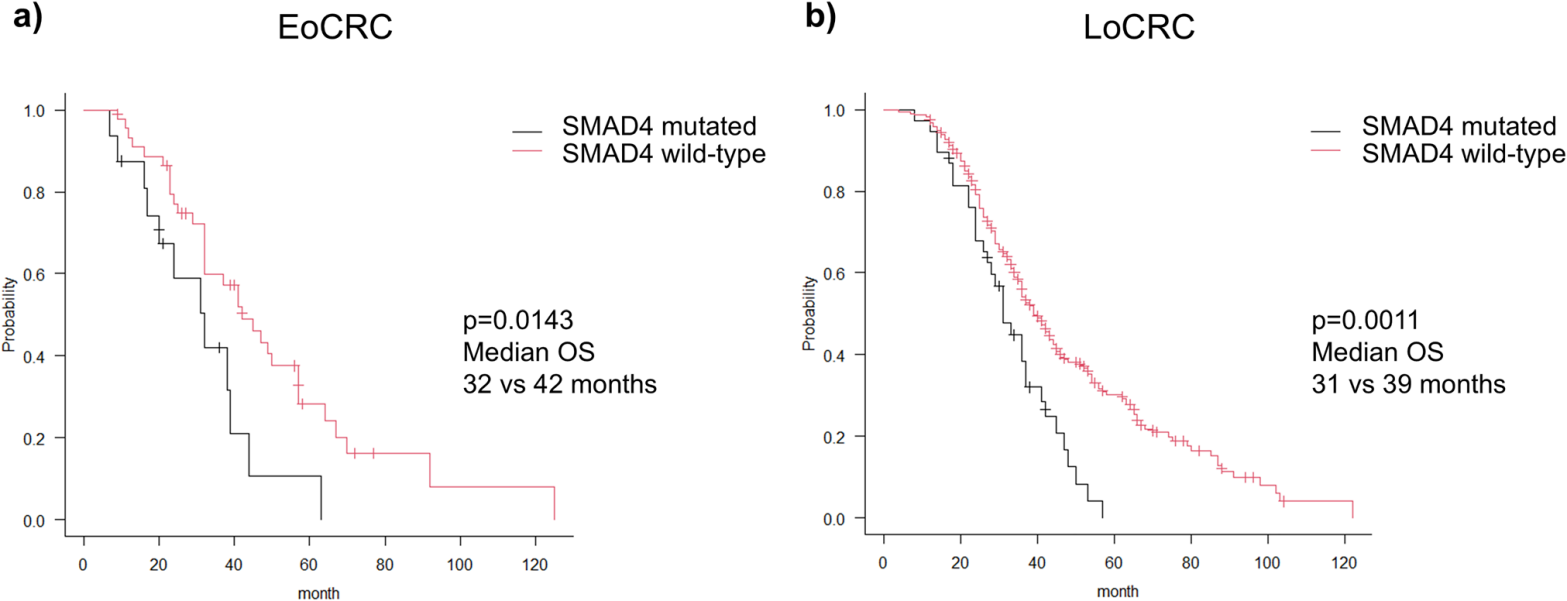
Overall survival analysis of patients with SMAD4 mutations. **a** Overall survival of SMAD4 mutation-positive and -negative patients in the EoCRC group. **b** Overall survival of SMAD4 mutation-positive and -negative patients in the LoCRC group

We next analyzed the clinical and prognostic significance of *SMAD4* and *FLT3* alterations in the EoCRC and LoCRC groups. *SMAD4* mutations were more frequently associated with colon tumors as opposed to rectal tumors in the EoCRC group, whereas no anatomical preference was observed in the LoCRC group (Table 2). Furthermore, *SMAD4* mutation-positive patients exhibited significantly worse prognosis compared to patients lacking *SMAD4* mutations in both groups (Fig. 4a, b). However, no significant difference was observed in the prognosis of EoCRC and LoCRC patients harboring *SMAD4* mutations.

**Table 2:** Comparison of clinicopathological features based on SMAD4 mutation status

	EoCRC		*p*-value	LoCRC		*p*-value
	SMAD4 mutated	SMAD4 wild-type		SMAD4 mutated	SMAD4 wild-type	
	*n* = 16	*n* = 45		*n* = 38	*n* = 218	
Sex						
Male	10	20	0.218	21	116	0.815
Location						
Right-side	1	4	0.743	10	57	0.983
Left-side	10	13	0.018	13	64	0.548
Rectum	5	28	0.034	15	97	0.566
Histology						
Differentiated	14	37	0.627	34	203	0.430
Initial stage						
IV	8	26	0.594	24	147	0.606

*EoCRC* early-onset colorectal cancer, *LoCRC* late-onset colorectal cancer

**Fig. 5:**
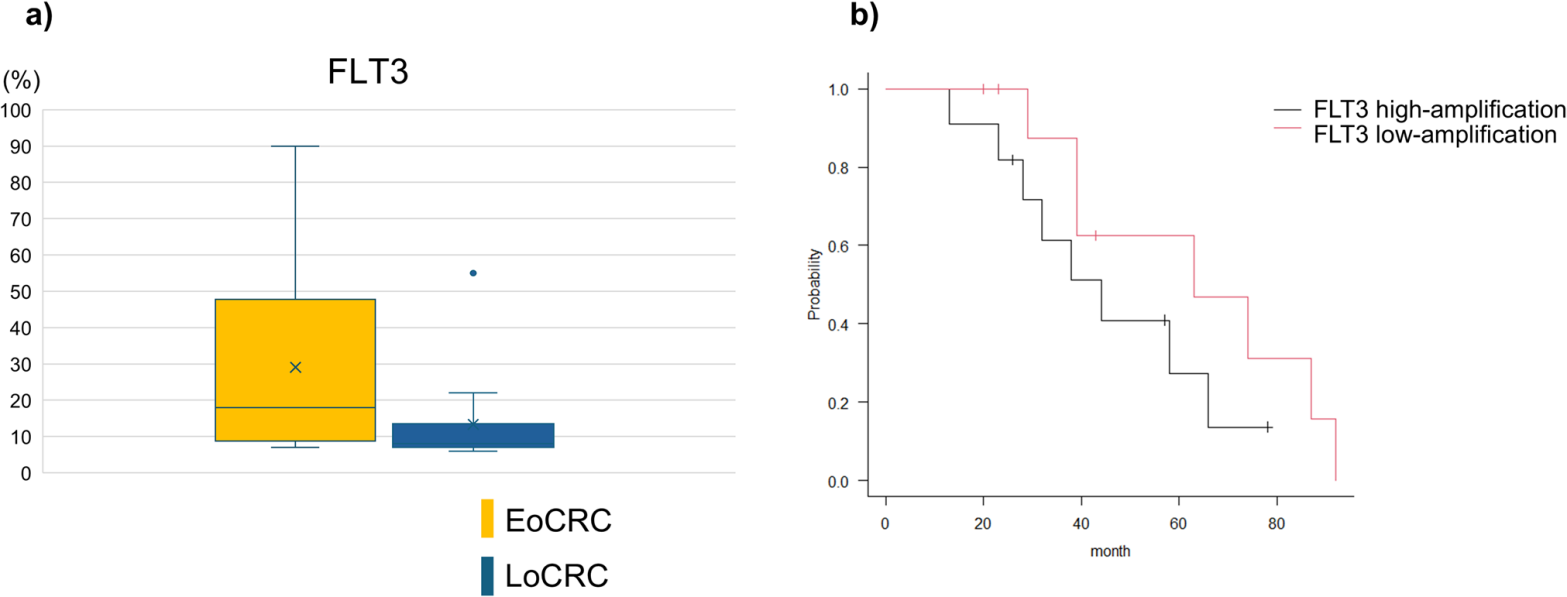
FLT3 amplification analysis. **a** Distribution of FLT3 copy number alterations in EoCRC and LoCRC patients. **b** Overall survival of patients with high-amplification and low-amplification of FLT3

Likewise, *FLT3* amplification did not show any significant association with patient characteristics or prognosis in either group (Supplementary Table 1). Notably, co-amplification of *CDK8* was identified in 75% of *FLT3* amplification-positive EoCRC patients and in 84.6% of LoCRC patients, and this association was statistically significant (*p* < 0.01). Furthermore, a higher proportion of EoCRC patients harbored ≥ 10 copies of *FLT3* compared to LoCRC patients (75% vs. 38.5%; Fig. 5a), although the difference was not significant (*p* = 0.086). Additionally, patients with higher copy number of *FLT3* tended to have worse prognosis compared to those with fewer copies (median OS; 44 vs. 63 months), albeit without statistical significance (*p* = 0.206; Fig. 5b). Interestingly, a more pronounced difference was observed between the prognosis of *FLT3*-high and *FLT3*-low amplification subgroups among patients with *FLT3* and *CDK8* co-amplification; however, the difference did not reach statistical significance (Supplementary Fig. 2).

## Discussion

This study is the first to evaluate the differences in genomic alterations between EoCRC and LoCRC in Japanese patients. Importantly, we found that genomic alterations such as *FLT3* and *CDK8*, which have not been reported previously, were significantly more common in EoCRC. These findings suggest that Japanese EoCRC may harbor unique genomic features compared with Western and Chinese cohorts.

Alterations in *PTCH1*, *KMT2A*, and *ZNF43*, which have been previously reported in EoCRC [17, 18], were not detected in this cohort. In contrast, *SMAD4*, *FLT3*, and *CDK8* were more frequently altered in EoCRC patients compared to the LoCRC patients. The discrepancies in genomic alterations observed in this study compared to previous reports are most likely due to ethnic background differences. In addition, the results may have been affected by variations in the detectable genes depending on the CGP testing platforms, as well as by differences in disease stage arising from patient selection criteria.

*SMAD4* mutations have been detected in 5–24.2% of CRC cases [24], particularly in the advanced stages, and more often in the colon relative to the rectum. Furthermore, these mutations associated with poor prognosis [24]. A previous study reported higher frequency of *SMAD4* alterations in younger CRC patients [25], which is consistent with our findings and suggests that *SMAD4* mutations may play a role in the pathogenesis of EoCRC. In addition, EoCRC patients with *SMAD4* alterations in our cohort demonstrated a significantly higher incidence of tumors in the colon -as opposed to the rectum- compared to the *SMAD4* alteration-negative patients, which is consistent with previous reports. Additionally, the presence of *SMAD4* alterations was significantly associated with poor prognosis. *SMAD4* is a tumor suppressor gene and acts as a mediator of the TGF-β signaling pathway, and *SMAD4* mutations contribute to tumor progression by disrupting cellular differentiation and proliferation. In fact, the TGF-β/SMAD4 signaling pathway has been implicated in CRC progression and warrants further investigation as a potential therapeutic target [26, 27]. Alterations in the TGF-β pathway were also more common in EoCRC patients, which corresponded to the higher incidence of *SMAD4* alterations in this group. Although EoCRC overall is more frequently located in the rectum, *SMAD4*-mutated EoCRC cases were more commonly found in the colon. Various molecular and biological differences have been reported between colon and rectal cancers [28], and *SMAD4* may play a more prominent role in the development of colon cancer in EoCRC, potentially through dysregulation of the TGF-β/SMAD4 signaling pathway. Further studies are needed to clarify the underlying mechanisms.

*FLT3* encodes a receptor tyrosine kinase (RTK) that plays a key role in hematopoiesis, and is overexpressed in MLL-rearranged acute lymphoblastic leukemia [29]. While *FLT3* amplification has been reported at low frequencies in CRC [30, 31], previous studies have suggested a potential inverse relationship between *FLT3* copy number and patient prognosis [31]. In this study, *FLT3* amplification was not significantly associated with prognosis, which may be attributed to the limited sample size. Furthermore, in line with previous findings, high- amplification of *FLT3* showed a trend towards worse overall survival. Altogether, these findings raise the possibility that *FLT3* may act as a potential oncogenic driver in CRC, thereby warranting further studies regarding its biological and therapeutic significance in EoCRC.

*CDK8* encodes a cell cycle-related kinase, and is frequently overexpressed in various cancers and has been identified as a prognostic biomarker in CRC [32]. In this study, cell cycle alteration was more common in the EoCRC group, likely due to the higher frequency of CDK8 amplification. Notably, both *FLT3* and *CDK8* alterations were amplification events, and co-amplification was observed in the majority of *FLT3*-amplified cases. This can be attributed to their genomic proximity, as *FLT3* and *CDK8* are both located on chromosome 13q12. A previous case report has also highlighted the amplification of both *FLT3* and *CDK8* in CRC [33]. The effects of FLT3 and CDK8 inhibitors on CRC cells have been investigated [34, 35]. Further functional analyses and therapeutic evaluations are necessary to explore the clinical utility of these alterations in precision medicine strategies for EoCRC.

There were several limitations in this study that ought to be considered. First, there was selection bias because we only included patients with CGP data. As CGP testing is typically performed for unresectable or recurrent patients, mutations in non-advanced CRC could not be analyzed. Therefore, our cohort is not entirely representative of all CRC patients. In addition, this study included only Japanese patients, which limits the generalizability of the findings. Second, the sample size, especially that of the EoCRC group, was small, thereby warranting further studies with larger sample size for more accurate results. Third, the use of different CGP testing platforms, each characterized by distinct gene panels, detection sensitivities, and analytical algorithms, may have introduced variability in the results and limited the direct comparability of genomic findings. Furthermore, we were not able to ascertain whether the genomic alterations detected with high frequency in the EoCRC group, particularly *FLT3* and *CDK8* amplification, are oncogenic. Therefore, functional studies are needed in future to elucidate their biological roles.

In conclusion, this study demonstrated that *SMAD4*, *FLT3*, and *CDK8* alterations are enriched in Japanese EoCRC patients, distinguishing them from previously reported Western and Chinese cohorts. Because these genes represent potentially actionable targets, our findings provide novel insights with important clinical implications.

## Supplementary Information

Below is the link to the electronic supplementary material.

Supplementary file1 (TIF 709 kb)

Supplementary file2 (TIF 592 kb)

Supplementary file3 (DOCX 15 kb)

## Data Availability

The data that support the findings of this study are available from the author, upon reasonable request.

## References

[1] Siegel RL, Torre LA, Soerjomataram I et al (2019) Global patterns and trends in colorectal cancer incidence in young adults. Gut 68(12):2179–2185. 10.1136/gutjnl-2019-319511 31488504 10.1136/gutjnl-2019-319511

[2] Sung JJY, Chiu HM, Jung KW et al (2019) Increasing trend in young-onset colorectal cancer in Asia: more cancers in men and more rectal cancers. Am J Gastroenterol 114(2):322–329. 10.14309/ajg.0000000000000133 30694865 10.14309/ajg.0000000000000133

[3] O’Sullivan DE, Sutherland RL, Town S et al (2022) Risk factors for early-onset colorectal cancer: a systematic review and meta-analysis. Clin Gastroenterol Hepatol 20(6):1229-1240.e5. 10.1016/j.cgh.2021.01.037 33524598 10.1016/j.cgh.2021.01.037

[4] Akimoto N, Ugai T, Zhong R et al (2021) Rising incidence of early-onset colorectal cancer - a call to action. Nat Rev Clin Oncol 18(4):230–243. 10.1038/s41571-020-00445-1 33219329 10.1038/s41571-020-00445-1 PMC7994182

[5] Zheng X, Hur J, Nguyen LH et al (2021) Comprehensive assessment of diet quality and risk of precursors of early-onset colorectal cancer. J Natl Cancer Inst 113(5):543–552. 10.1093/jnci/djaa164 33136160 10.1093/jnci/djaa164 PMC8096368

[6] Hussan H, Patel A, Ma J et al (2023) Historical obesity and early-onset cancers: a propensity-weighted analysis of the National Health and Nutrition Examination Survey. Dig Dis Sci. 10.1007/s10620-023-08194-8 38030832 10.1007/s10620-023-08194-8

[7] Wu CW, Lui RN (2022) Early-onset colorectal cancer: current insights and future directions. World J Gastrointest Oncol 14(1):230–241. 10.4251/wjgo.v14.i1.230 35116113 10.4251/wjgo.v14.i1.230 PMC8790420

[8] Carethers JM (2015) Screening for colorectal cancer in African Americans: determinants and rationale for an earlier age to commence screening. Dig Dis Sci 60(3):711–721. 10.1007/s10620-014-3443-5 25540085 10.1007/s10620-014-3443-5 PMC4369177

[9] Mármol I, Sánchez-de-Diego C, PradillaDieste A et al (2017) Colorectal carcinoma: a general overview and future perspectives in colorectal cancer. Int J Mol Sci 18(1):197. 10.3390/ijms18010197 28106826 10.3390/ijms18010197 PMC5297828

[10] Mauri G, Sartore-Bianchi A, Russo AG et al (2019) Early-onset colorectal cancer in young individuals. Mol Oncol 13(2):109–131. 10.1002/1878-0261.12417 30520562 10.1002/1878-0261.12417 PMC6360363

[11] Khan M, Korphaisarn K, Saif A et al (2017) Early-onset signet-ring cell adenocarcinoma of the colon: a case report and review of the literature. Case Rep Oncol Med 2017:2832180. 10.1155/2017/2832180 28326211 10.1155/2017/2832180 PMC5343248

[12] Chang DT, Pai RK, Rybicki LA et al (2012) Clinicopathologic and molecular features of sporadic early-onset colorectal adenocarcinoma: an adenocarcinoma with frequent signet ring cell differentiation, rectal and sigmoid involvement, and adverse morphologic features. Mod Pathol 25(8):1128–1139. 10.1038/modpathol.2012.61 22481281 10.1038/modpathol.2012.61

[13] Wells K, Wise PE (2017) Hereditary colorectal cancer syndromes. Surg Clin North Am 97(3):605–625. 10.1016/j.suc.2017.01.009 28501250 10.1016/j.suc.2017.01.009

[14] Takada K, Hotta K, Imai K et al (2022) Favorable survival after screening for young-onset colorectal cancer: benefits of screening in young adults. Dis Colon Rectum 65(8):996–1004. 10.1097/DCR.0000000000002106 34856591 10.1097/DCR.0000000000002106

[15] Okagawa Y, Seto K, Yoshida K et al (2025) Clinicopathological features of early-onset colorectal cancer in Japanese patients: a single-center retrospective study. BMC Gastroenterol 25(1):156. 10.1186/s12876-025-03725-1 40069641 10.1186/s12876-025-03725-1 PMC11899674

[16] Li Q, Yu M, Lv H et al (2023) Burden of early-onset colorectal cancer along with attributable risk factors from 1990 to 2019: a comparative study between China and other G20 countries. BMC Public Health 23(1):1463. 10.1186/s12889-023-16407-y 37525147 10.1186/s12889-023-16407-y PMC10391986

[17] Li P, Meng Q, Xue Y et al (2022) Comprehensive genomic profiling of colorectal cancer patients reveals differences in mutational landscapes among clinical and pathological subgroups. Front Oncol 10(12):1000146 10.3389/fonc.2022.1000146 PMC9685809 36439454

[18] Busico A, Gasparini P, Rausa E et al (2024) Molecular profiling of pediatric and young adult colorectal cancer reveals a distinct genomic landscapes and potential therapeutic avenues. Sci Rep 14(1):13138 38849509 10.1038/s41598-024-64149-7 PMC11161608

[19] Horie S, Saito Y, Kogure Y et al (2024) Pan-cancer comparative and integrative analyses of driver alterations using Japanese and international genomic databases. Cancer Discov 14(5):786–803 38276885 10.1158/2159-8290.CD-23-0902

[20] Naito Y, Aburatani H, Amano T et al (2021) Clinical practice guidance for next-generation sequencing in cancer diagnosis and treatment (edition 2.1). Int J Clin Oncol 26(2):233–283 33249514 10.1007/s10147-020-01831-6 PMC7819967

[21] Kikuchi J, Ohhara Y, Takada K et al (2021) Clinical significance of comprehensive genomic profiling tests covered by public insurance in patients with advanced solid cancers in Hokkaido, Japan. Jpn J Clin Oncol 51(5):753–761 33532831 10.1093/jjco/hyaa277

[22] Serizawa M, Mizuguchi M, Urakami K et al (2021) JCGA: the Japanese version of the Cancer Genome Atlas and its contribution to the interpretation of gene alterations detected in clinical cancer genome sequencing. Hum Genome Var 8(1):38 34588443 10.1038/s41439-021-00170-w PMC8481308

[23] Kanda Y (2013) Investigation of the freely available easy-to-use software ‘EZR’ for medical statistics. Bone Marrow Transplant 48(3):452–458 23208313 10.1038/bmt.2012.244 PMC3590441

[24] Fang T, Liang T, Wang Y et al (2021) Prognostic role and clinicopathological features of SMAD4 gene mutation in colorectal cancer: a systematic review and meta-analysis. BMC Gastroenterol 21(1):297 34301194 10.1186/s12876-021-01864-9 PMC8299661

[25] Puccini A, Lenz HJ, Marshall JL et al (2019) Impact of patient age on molecular alterations of left-sided colorectal tumors. Oncologist 24(3):319–326 30018131 10.1634/theoncologist.2018-0117 PMC6519749

[26] Ma C, Liu M, Feng W et al (2023) Loss of SETD2 aggravates colorectal cancer progression caused by SMAD4 deletion through the RAS/ERK signalling pathway. Clin Transl Med 13(11):e1475 37962020 10.1002/ctm2.1475 PMC10644329

[27] Yang M, Li D, Jiang Z et al (2022) TGF-β-induced FLRT3 attenuation is essential for cancer-associated fibroblast-mediated epithelial-mesenchymal transition in colorectal cancer. Mol Cancer Res 20(8):1247–1259 35560224 10.1158/1541-7786.MCR-21-0924

[28] Surakhy M, Matheson J, Barnes DJ et al (2025) Smad4 and TGFβ1 dependent gene expression signatures in conditional intestinal adenoma, organoids and colorectal cancer. Sci Rep 15(1):16330 40348815 10.1038/s41598-025-00908-4 PMC12065906

[29] Armstrong SA, Kung AL, Mabon ME et al (2003) Inhibition of FLT3 in MLL. Validation of a therapeutic target identified by gene expression based classification. Cancer Cell 3(2):173–183 12620411 10.1016/s1535-6108(03)00003-5

[30] Cancer Genome Atlas Network (2012) Comprehensive molecular characterization of human colon and rectal cancer. Nature 487(7407):330–337 22810696 10.1038/nature11252 PMC3401966

[31] Hasegawa H, Taniguchi H, Nakamura Y et al (2021) Fms-like tyrosine kinase 3 (FLT3) amplification in patients with metastatic colorectal cancer. Cancer Sci 112(1):314–322 33075166 10.1111/cas.14693 PMC7780005

[32] Wang D, Zhou Y, Hua L et al (2022) CDK3, CDK5 and CDK8 proteins as prognostic and potential biomarkers in colorectal cancer patients. Int J Gen Med 27(15):2233–2245 10.2147/IJGM.S349576 PMC8893271 35250301

[33] Aydın E, Tokat ÜM, Adibi A et al (2024) Case report: Precision guided reactive cancer management: molecular complete response in heavily pretreated metastatic CRC by dual immunotherapy and sorafenib. Front Oncol 1(14):1405170 10.3389/fonc.2024.1405170 PMC11246968 39011472

[34] Lin D, Xu Y, Zhan H et al (2025) Targeting the ZMYM2-ANXA9 axis with FLT3 inhibitor G749 overcomes oxaliplatin resistance in colorectal cancer. Biomedicines 13(5):1247 40427072 10.3390/biomedicines13051247 PMC12108716

[35] Xi M, Chen T, Wu C et al (2019) CDK8 as a therapeutic target for cancers and recent developments in discovery of CDK8 inhibitors. Eur J Med Chem 15(164):77–91 10.1016/j.ejmech.2018.11.076 30594029

