# Supplementary figures and images for: Characterization of the mutational landscapes in Japanese patients with early-onset colorectal cancer from comprehensive genomic profiling data
Source: Int J Clin Oncol. 2025 Oct 11;30(12):2596–604. doi: 10.1007/s10147-025-02889-w (PMC12644146; doi:10.1007/s10147-025-02889-w)

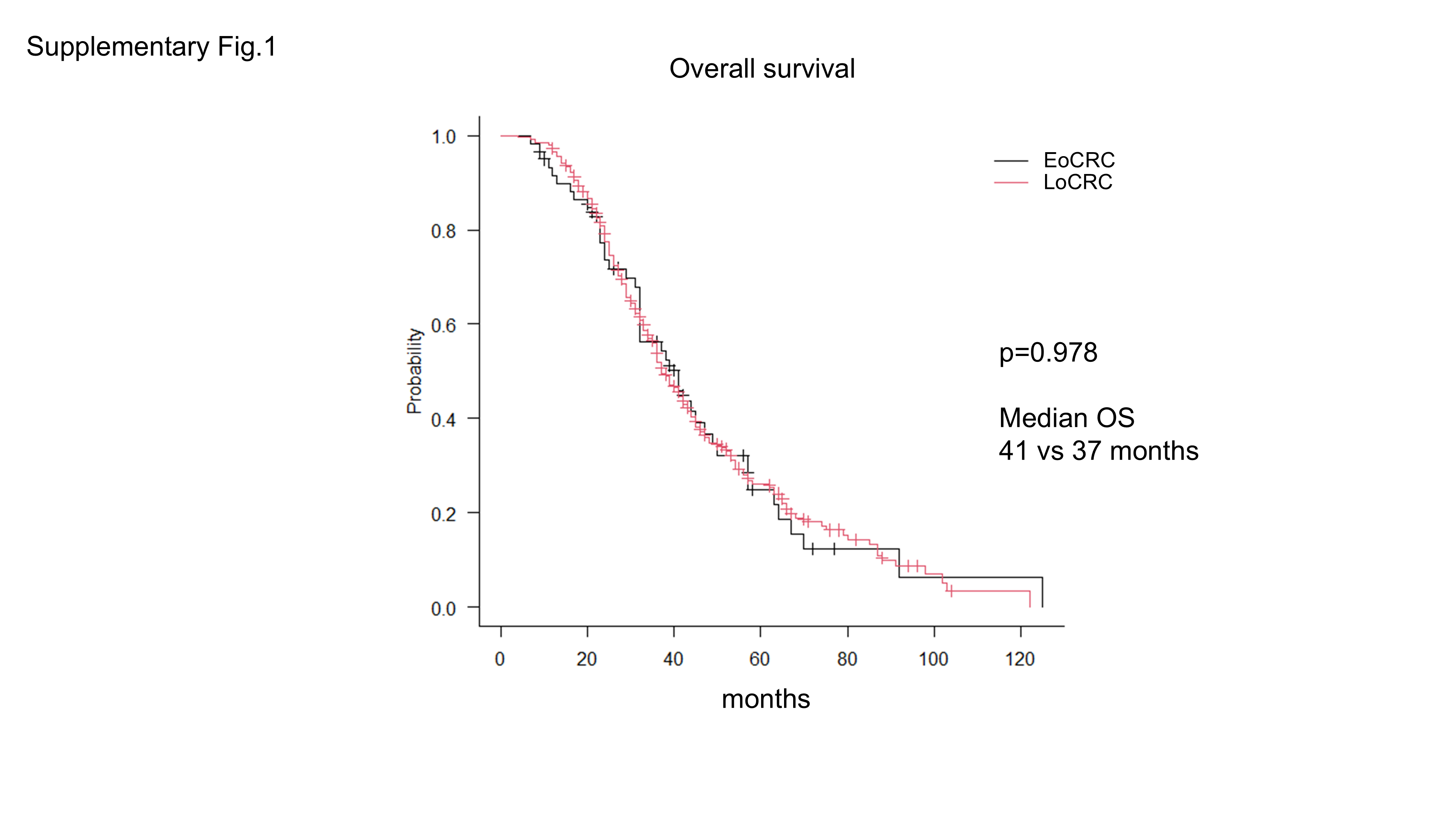

Supplement: Supplementary file 1 — Supplementary file1 (TIF 709 kb) [file 10147_2025_2889_MOESM1_ESM.tif]

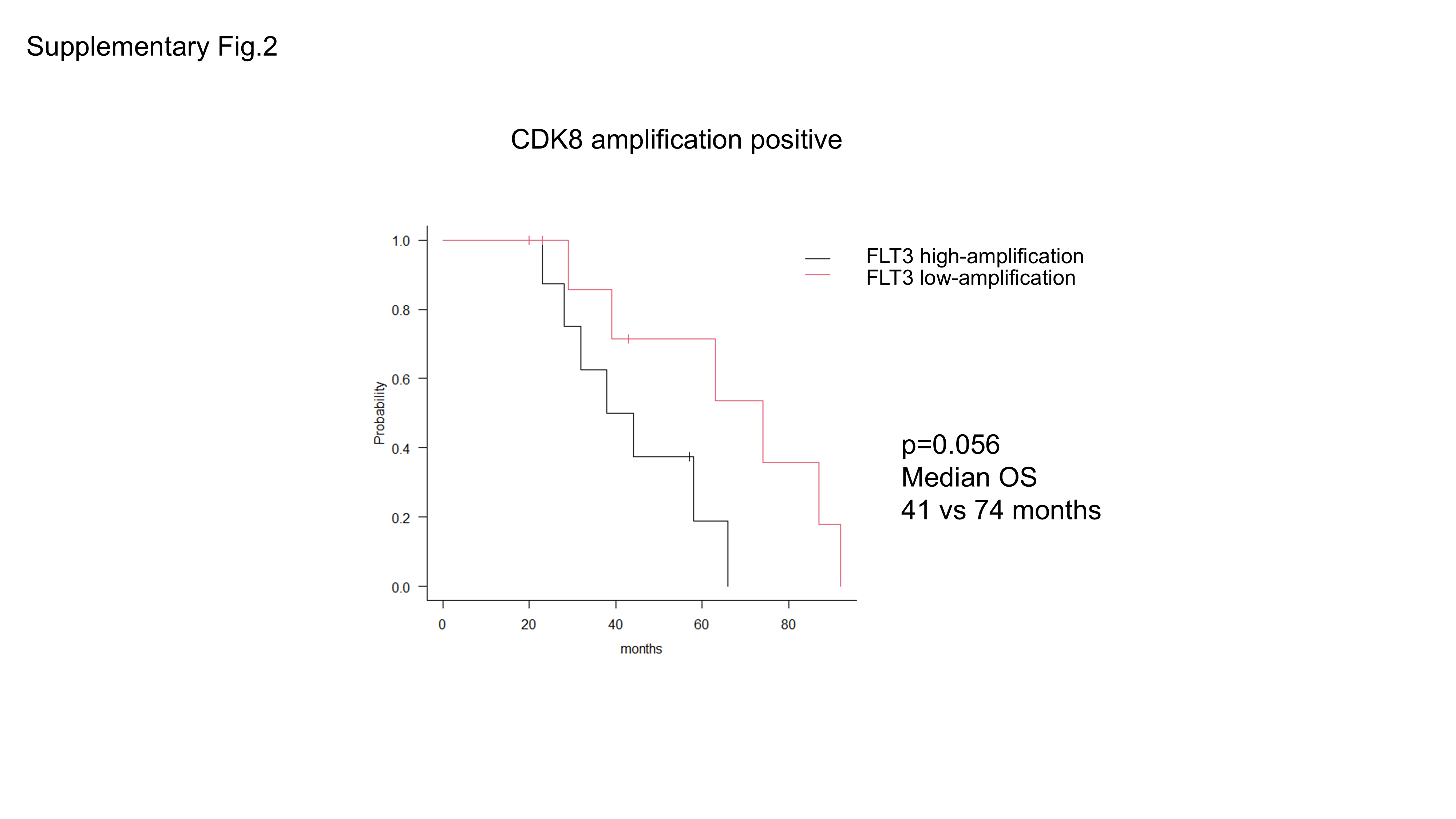

Supplement: Supplementary file 2 — Supplementary file2 (TIF 592 kb) [file 10147_2025_2889_MOESM2_ESM.tif]
