# Supplementary material for: Characterization of the mutational landscapes in Japanese patients with early-onset colorectal cancer from comprehensive genomic profiling data
Source: Int J Clin Oncol. 2025 Oct 11;30(12):2596–604. doi: 10.1007/s10147-025-02889-w (PMC12644146; doi:10.1007/s10147-025-02889-w)
Supplement: Supplementary file 3 — Supplementary file3 (DOCX 15 kb) [file 10147_2025_2889_MOESM3_ESM.docx]

Supplementary Table.1

|  | EoCRC | | p-value |  | LoCRC | | p-value |
| --- | --- | --- | --- | --- | --- | --- | --- |
|  | FLT3 mutated | FLT3 wild-type |  |  | FLT3 mutated | FLT3 wild-type |  |
|  | n=8 | n=51 |  |  | n=13 | n=243 |  |
| Sex |  |  |  |  |  |  |  |
| male | 3 | 27 | 0.421 |  | 8 | 129 | 0.552 |
| Location |  |  |  |  |  |  |  |
| Right-side | 0 | 5 | 0.359 |  | 2 | 65 | 0.365 |
| Left-side | 3 | 20 | 0.927 |  | 5 | 72 | 0.45 |
| Rectum | 5 | 28 | 0.69 |  | 6 | 106 | 0.858 |
| Histology |  |  |  |  |  |  |  |
| Differentiated | 8 | 43 | 0.232 |  | 13 | 224 | 0.296 |
| Initial Stage |  |  |  |  |  |  |  |
| IV | 5 | 29 | 0.766 |  | 8 | 163 | 0.703 |

EoCRC; early-onset colorectal cancer, LoCRC; late-onset colorectal cancer
